# 19-month-old Girl with Seizure

**DOI:** 10.5811/cpcem.48773

**Published:** 2026-04-07

**Authors:** Julie Kurek, Cheyenne Falat, Laura J. Bontempo, J. David Gatz

**Affiliations:** * University of Maryland Medical Center, Department of Emergency Medicine and Pediatrics, Baltimore, Maryland; † University of Maryland School of Medicine, Department of Emergency Medicine, Baltimore, Maryland

## Abstract

Pediatric seizures are an alarming presentation to the emergency department (ED) that can be caused by a multitude of etiologies. It is important to differentiate life-threatening conditions from more benign causes. A 19-month-old girl presented to the ED after a witnessed seizure. This case offers a differential diagnosis for pediatric seizures and uses history, exam, laboratory findings, and imaging to hone the differential in the ED setting. The surprising final diagnosis and case outcome are then revealed and discussed.

## CASE PRESENTATION (DR. KUREK)

A 19-month-old girl was brought into the emergency department (ED) by emergency medical services (EMS) for suspected seizure. The EMS personnel were called to a daycare facility where the child was being fed oatmeal when a daycare worker noticed that she suddenly became unresponsive and had full body shaking. The full body shaking lasted approximately one minute before spontaneously stopping. On their arrival, EMS responders found the patient to be minimally responsive to painful stimuli (i.e., the placement of an intravenous line).

Upon arrival to the ED, the patient had become awake and alert, but she was unable to provide any significant history due to her age. Her parents noted that the patient had been acting like her normal self and at her baseline health, other than seeming more tired than usual. Otherwise, her review of systems was negative for any other symptoms. She had no significant past medical history, including no prior history of seizures. She had no past surgical history. She was born full term with no acute complications. She was unvaccinated. She was not taking any medications and had no known drug allergies. Her family did not have any significant medical history. Her parents reported that the patient’s siblings had rhinorrhea from a suspected viral upper respiratory infection.

On initial presentation, the patient’s vital signs were as follows: temperature, 37.3 °C; heart rate, 137 beats per minute; blood pressure, 120/62 millimeters of mercury; respiratory rate, 40 breaths per minute; and oxygen saturation 100% on room air. Her weight was 11.4 kilograms. On examination, the patient had a normal appearance and was not in acute distress. Her head was normocephalic and atraumatic. External ears appeared normal bilaterally, as did her nose. There was some dried food around her mouth, but her oropharynx was clear and her oral mucous membranes were moist. Her pupils were equal and reactive, and she appeared to demonstrate normal extraocular movements. Her heart had a regular rhythm, moderate tachycardia, and no murmurs. Her lungs were clear to auscultation bilaterally. Her abdomen was soft, non-distended, and non-tender. Her extremities showed no edema or tenderness. Her skin was warm and dry. Neurologically, she was awake and alert, moving all her extremities and reaching for her parents. There were no apparent focal deficits. Further musculoskeletal examination and ambulation were not performed as part of the initial assessment.

Laboratory studies (Table) indicated that her complete blood count was notable for mild normocytic anemia. Her basic metabolic panel was notable for hyponatremia and slightly low bicarbonate. The liver function tests were notable for an elevated aspartate aminotransferase with a normal alanine aminotransferase level, along with an elevated alkaline phosphatase. The potassium, magnesium, and calcium values were not available due to hemolysis. A respiratory viral panel was positive for rhino enterovirus. A urine toxicology screen was negative for any tested substances.

While in the ED, the patient experienced a second seizure complicated by oxygen saturation levels of 70–80%. She was provided supplemental oxygen via a non-rebreather mask at 15 liters per minute, and the seizure activity self-terminated. The patient had a third seizure approximately 30 minutes later, which resolved after 2 mg of intravenous midazolam.

**Image 1:**
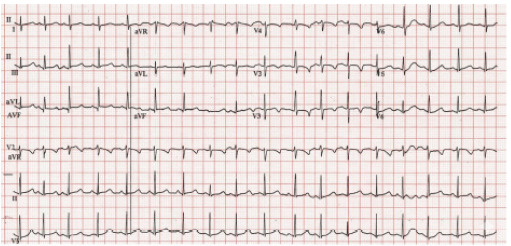
Electrocardiogram of a 19-month-old girl with seizure. (To improve readability, the original image was enhanced using Google’s large-language model [Imagen], gemini.google.com, 10/2/2025).

**Image 2:**
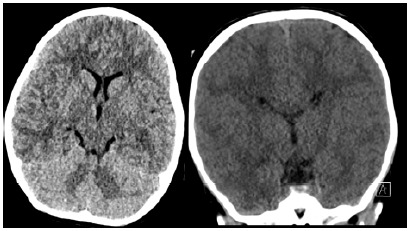
Representative axial and coronal images from a computed tomography head of a 19-month-old girl with seizure.

Due to the repeat seizure activity, an electrocardiogram (Image 1) and an unremarkable computed tomography (CT) of the head (Image 2) were obtained. Subsequent to these results, a test was ordered, and a diagnosis was made.

## CASE DISCUSSION (DR. FALAT)

I am an attending physician at an academic adult ED, where I provide medical care for adults > 21 years of age. So, when I was presented with this case of a 19-month-old child with a seizure, I had to fall back on my pediatric basics to start to extract what I felt was pertinent information from the presentation. While mentally retaining information I felt was important as I worked my way through this case, I also mentally “discarded” information I did not feel to be pertinent. This way, I could stay focused, trusting I’d find a framework for my differential along the way.

Upon reviewing the chief complaint, I felt that both the child’s age and the presentation of seizure were important. Reading through the history of present illness, I also felt it was important to note that the suspected seizure lasted for approximately one minute, that the child appeared postictal and “sleepy” afterward, and that the child had been atypically tired for the preceding few days. Information I chose to mentally discard included the following: the child had been eating oatmeal prior to her seizure; EMS did not administer any medications; the patient had stable vital signs for transport; the child was otherwise in her usual state of health; and she had siblings with rhinorrhea.

There was not much that stood out within the past history and, therefore, I mentally discarded that the child had an uncomplicated birth at full term, did not have any medical or surgical history, was not on medications, and had no allergies. However, there was something significant that stuck out to me at this stage of the presentation—the child was unvaccinated. This spurred my interest. Why did the parents choose not to vaccinate their child? Were there other religious, social, or nutritional issues that would factor into the health of this child? Were the parents disregarding other evidence-based recommendations for their child’s health? This vital piece of information stuck with me as I went through the remainder of this case.

As I dissected the review of systems, I did not find that it contributed to building my differential diagnosis, aside from reassuring me against some of the serious etiologies of pediatric seizures. For instance, the absence of vomiting, headaches, wounds, and ecchymosis significantly lowered the likelihood of intracranial hemorrhage or non-accidental trauma.

I was happy to find that the child’s physical examination was also reassuring. While her systolic blood pressure was borderline high for her age, the remainder of her vitals (including heart rate, respiratory rate, and temperature) were all within age-adjusted normal vital sign ranges. It is typical for patients to have slight elevations in their blood pressure and heart rate when under stress or after stressful events, such as a seizure, so I chose not to mentally retain any of the vital signs. Pertinent physical examination findings included the absence of neurologic deficits, the movement of all extremities, the full extraocular movements, the equally round and reactive pupils, and the “sleepy” but increasingly alert mental status. These findings also reassured me against a space-occupying lesion or cerebrovascular accident as the etiology of seizure, as I would anticipate that with these etiologies there would be persistent focal findings on the examination. I chose to mentally discard the remainder of the physical examination, while noting that the child did not appear dehydrated, did not appear to have external evidence of trauma or injuries (warm and well perfused skin, normocephalic and atraumatic head, and normal musculoskeletal range of movement), and did not demonstrate cardiopulmonary distress.

The laboratory values for the patient’s potassium, magnesium, and calcium were not immediately available due to hemolysis of the sample. In the absence of another etiology of the seizure to this point, I suspected these electrolytes would play a vital role in narrowing my differential diagnosis. Additionally, the patient did not fit any classic toxidrome, and the urine toxicology was negative for all substances tested; therefore, a toxicologic etiology of the seizure was much less likely. The normal blood glucose eliminated hypoglycemia from my differential, and I was reassured to find only mild anemia and hyponatremia, with no acute kidney injury. The child did test positive for rhino/enterovirus, but this is common for young children in daycare. This, therefore, did not strike me as terribly abnormal, but perhaps it explained why the child had appeared tired over the prior few days.

After the child had recurrent seizures while in the ED, additional testing was done. The normal head CT confirmed my low suspicion for intracranial hemorrhage or a space-occupying lesion. The electrocardiogram (ECG), however, was much less reassuring. I calculated the corrected QT (QTc) interval as approximately 500 milliseconds. This was not normal and made me think back to the child’s missing electrolytes, as hypokalemia, hypomagnesemia, and hypocalcemia can all cause a prolonged QTc interval.^1^

As I started reviewing this case in its totality, I still needed to figure out what had caused this unvaccinated, 19-month-old child to have multiple seizures of approximately one-minute duration each, with notable absence of neurologic deficits and improving alertness between seizures, whose workup included a normal blood glucose and normal head CT, but with an abnormal ECG demonstrating a prolonged QTc interval with unknown electrolytes.

As an emergency physician, I could not stop thinking about this abnormal ECG. While discussing a clinicopathologic conference case or working an ED shift, I must make some assumptions along the way. I assumed that an abnormal ECG would surely matter for this child. This now meant that I not only had one differential for pediatric seizures to work through, but a second differential for prolonged QTc intervals to also work through!

I realized that these two daunting differentials could suddenly become much easier to work through by looking for overlapping etiologies. I found three clear overlapping etiologies—hypocalcemia, trauma (with intracranial hemorrhage), and stroke. The child’s neurological examination and head CT quickly ruled out the latter two, which left me with a diagnosis of hypocalcemia!

Suddenly, everything in this presentation fell together. Seizures are considered a classic symptom of severe hypocalcemia, and patients with hypocalcemia are at risk of developing prolonged QTc intervals.^2^,^3^ After reviewing the literature on hypocalcemia, I was reminded that it is commonly related to vitamin D deficiency, known as rickets in children.^4^ Then I thought back to the child’s unvaccinated status and uncovered another potential overlap—perhaps the unvaccinated status and presumed Vitamin D deficiency shared a common root in a strict, plant-based diet, as some vaccines are grown in eggs or contain gelatin and are, therefore, declined by vegans. Children who adhere to strict, plant-based diets may be at risk for deficiencies in proteins, iron, zinc, selenium, calcium, riboflavin, Vitamin A, Vitamin D, Vitamin B12, and essential fatty acids.^5^

Thus, what started as an intimidating journey into the land of pediatric emergency medicine ended in a sure destination—hypocalcemia-related seizure in the setting of dietary Vitamin D deficiency, for which I’d start by ordering ionized calcium and Vitamin D levels.

## CASE OUTCOME (DR. KUREK)

Serum calcium and ionized calcium levels returned at 5.7 milligrams per deciliter (mg/dL) (normal value 9.0–11.0 mg/dL) and 0.66 millimoles per liter (mmol/L) (1.15–1.29 mmol/L), respectively, causing immediate suspicion for hypocalcemia-induced seizures. The patient was treated in the pediatric ED with 60 mg/kg of calcium gluconate and admitted to the pediatric intensive care unit for further management and care. Pediatric endocrinology was consulted and started the patient on calcium carbonate 50mg/kg/day divided into doses every six hours; calcitriol 0.25 micrograms daily; and ergocalciferol 5,000 international units daily. An ionized calcium level was trended every four hours, and a comprehensive metabolic panel was obtained every eight hours. Pediatric endocrinology also ordered vitamin D levels, and results showed significant vitamin D deficiency. Vitamin D2 25 hydroxy, Vitamin D 25 hydroxy, and Vitamin D3 25 hydroxy were all < 4 nanograms per milliliter (mL). An obtained parathyroid (PTH) level was significantly elevated at 731 picograms/mL.

**Image 3:**
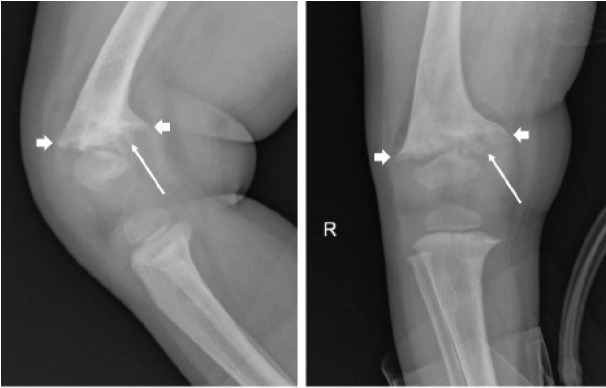
Right knee radiograph of a 19-month-old girl with seizure demonstrating fraying (long arrows) and splaying (short arrows) of the metaphysis.

Further discussion with family revealed that the patient’s family was strictly vegan and, as a result, the patient was also following a vegan diet. Radiographs of the bilateral wrists and knees were obtained to assess for rickets and demonstrated classic findings of “fraying”/“splaying” of the metaphysis (Image 3). The patient’s calcium levels eventually normalized enough after a week in the hospital to be discharged home. As an outpatient she continued a regimen of calcitriol, calcium carbonate, and vitamin D supplementation. The patient was scheduled to follow up with endocrinology for repeat blood work but was unfortunately lost to follow-up.

## RESIDENT DISCUSSION (DR. KUREK)

First-time seizure is a common presentation to the pediatric ED. As with any resuscitation, it is important to first secure the “ABCs”: ensuring the patient is maintaining their own airway; oxygenating; and maintaining a pulse and appropriate blood pressure. A point-of-care blood glucose to exclude hypoglycemia is a reasonable “D” (for dextrose) as a part of this initial assessment. Physicians should rapidly follow this initial stabilization with a thoughtful consideration of the differential, which can be quite broad and include everything from trauma, infection or intoxication to stroke, metabolic disorders, or a brain tumor.

Ultimately this patient suffered seizures secondary to a severe calcium deficiency. Common symptoms of hypocalcemia are muscle cramping, muscle aches, and numbness/tingling. Classic physical exam findings include Chvostek sign and Trousseau sign. Chvostek sign involves twitching of the facial muscles upon tapping the lateral side of the face in front of the ear overlying the facial nerve. Trousseau sign manifests as distal flexion of the arm with inflation of a blood pressure cuff. More severe levels of hypocalcemia cause patients to become altered, have seizures, hallucinate, or even have cardiac arrythmias due to a prolonged QTc.^6^

Patients may have hypocalcemia for multiple reasons. These include decreased absorption (vitamin D deficiency, hypoparathyroidism), increased excretion (renal failure, alcoholism), drug-induced (e.g., loop diuretics), and miscellaneous other causes such as rhabdomyolysis and sepsis. Massive blood transfusion is a possible iatrogenic cause of hypocalcemia as the citrate preservative binds to ionized calcium, lowering the amount of calcium available in the blood stream.^6^

Diagnostic studies to consider when working someone up with suspected hypocalcemia include serum calcium and ionized calcium levels to confirm the diagnosis, and a serum PTH level. Additional recommended studies include serum albumin, alkaline phosphatase, creatinine, magnesium, and phosphate, as these can help identify the etiology of the hypocalcemia. For example, abnormal liver function or renal function could explain why calcium is low due to their importance in the processing of vitamin D and thus calcium absorption. Concerns of vitamin D deficiency or malabsorption can be investigated by measuring the vitamin D metabolites calcidiol (25-hydroxyvitamin D) and calcitriol (1,25 dihydroxyvitamin D). A low albumin level can affect the measured calcium level, and physicians may need to perform a calcium correction calculation (listed below), although some have questioned the value/accuracy of this practice.^7^


Corrected Calcium=(0.8*(Normal Albumin-Patient’s Albumin))+Serum Calcium

Vitamin D plays a critical role in the absorption of calcium. Normally the body will release more PTH in response to low calcium levels. This PTH impacts the kidneys, intestines, and bones. PTH will stimulate the bones to release calcium and stimulate the kidneys to resorb more calcium. Additionally, it prompts the kidneys to make the active version of vitamin D. Vitamin D (ergocalciferol and cholecalciferol) is transformed into calcidiol (25-hydroxyvitamin D) in the liver. Then the calcidiol will be transformed into an active form – 1,25 dihydroxyvitamin D (also called calcitriol). Calcitriol is the form of vitamin D that assists the gut with absorbing calcium.^8^

Understanding the interplay of these factors can allow clinicians to identify specific pathology causing hypocalcemia by the relative level of each item. For example, in patients with hypoparathyroidism, lab values typically show a low PTH with normal alkaline phosphatase and normal calcidiol (25-hydroxyvitamin D). For patients with vitamin D deficiency, PTH will be elevated with low calcidiol (25-hydroxyvitamin D) and a high alkaline phosphatase. For a patient with PTH resistance, PTH will be elevated, calcium low with normal levels of calcidiol (25-hydroxyvitamin D) and normal to low levels of 1,25 dihydroxyvitamin D called calcitriol.^9^

Immediate treatment is to correct serum calcium levels with calcium gluconate (if only peripheral intravenous access is available) and/or calcium chloride (if central line access is available). The goal is to achieve an ionized calcium level around one mmol/liter. Physicians should check ionized calcium levels every four to six hours. If there is concern for rickets or other chronic nutritional deficiencies, physicians should also replete the vitamin D and calcium levels.

This case notably involved a child receiving a strict vegan diet alongside the rest of her family. Vegan diets exclude any animal-based products – including cow milk and can result in low vitamin D and calcium levels. Similarly, there are case reports of infants having seizures due to the use of homemade vegan formula and from being breast fed by mothers who themselves are vitamin D deficient.^10^,^11^ There are, however, a variety of foods available today that are vitamin D and calcium fortified. Additionally, vegan sources of calcium include foods such as vegetables like spinach and kale, legumes like chickpeas, fruit like pears and oranges, and calcium-fortified almond milk. Due to the risk of malnutrition and inadequate development, the American Academy of Pediatrics states that many non-dairy milk alternatives do not contain enough vitamins or nutrients for children under the age of one. It is recommended that a child under the age of one be primarily fed with formula or breast milk. For children older than one year of age, parents and children need to have a solid understanding of nutritional components of vegan food or what food items are fortified.^12^,^13^

Overall, calcium levels are dependent on a variety of factors from gut absorption to parathyroid disorders to vitamin D levels.

## FINAL DIAGNOSIS

Seizure due to severe hypocalcemia and vitamin D deficiency in a child receiving a vegan diet.

## KEY TEACHING POINTS

1. Keep a broad differential for seizures in children.
2. Take a detailed social history for children. Diet in young children is very important and deficient diets can have a vast effect on health.
3. If you are concerned for hypocalcemia in a patient, obtain a calcium level, ionized calcium, albumin level, and PTH for initial evaluation.

## Figures and Tables

**Table:** Initial laboratory results of a 19-month-old girl who presented to the emergency department with seizure.

Test	Patient value	Normal value
Complete Blood Count		
White Blood Cell	10.5 K/mcL	4.5 – 11 K/mcL
Hemoglobin	10.3 g/dL	11.9 – 15.7 g/dL
Hematocrit	32.8%	35.0 – 45.0%
Platelets	289 K/mcL	150 – 350 K/mcL
Complete Metabolic Panel		
Sodium	130 mmol/L	136 – 145 mmol/L
Potassium	***	3.5 – 5.1 mmol/L
Chloride	103 mmol/L	98 – 107 mmol/L
Bicarbonate	20 mmol/L	21 – 30 mmol/L
Blood urea nitrogen	12 mg/dL	7 – 17 mg/dL
Creatinine	0.16 mg/dL	0.52 – 1.04 mg/dL
Glucose	72 mg/dL	70 – 100 mg/dL
Albumin	3.9 g/dL	3.2 – 4.6 g/dL
Total bilirubin	0.3 mg/dL	0.3 – 1.2 mg/dL
Aspartate aminotransferase	43 units/L	14 – 36 units/L
Alanine aminotransferase	20 units/L	0 – 34 units/L
Alkaline phosphatase	551 units/L	38 – 126 units/L
Additional Labs		
Calcium	***	9.0 – 11.0 mg/dL
Ionized Calcium	***	1.15 – 1.29 mmol/L
Magnesium	***	1.6 – 2.6 mg/dL
Urine toxicology screen		
Fentanyl	Negative	Negative
Oxycodone	Negative	Negative
Amphetamine	Negative	Negative
Barbiturate	Negative	Negative
Benzodiazepine	Negative	Negative
Cannabinoid	Negative	Negative
Cocaine Metabolite	Negative	Negative
Methadone	Negative	Negative
Opiate	Negative	Negative
Phencyclidine	Negative	Negative

*** indicates a hemolyzed sample.

*dL*, deciliter; *g*, grams; *K*, thousands; *L*, liter; *mcL*, microliter; *mg*, milligram; *mmol*, millimole.

## References

[1] Khan IA (2002). Long QT syndrome: diagnosis and management. Am Heart J.

[2] Wynne Z, Falat C (2023). Disorders of calcium and magnesium. Emerg Med Clin North Am.

[3] Bove-Fenderson E, Mannstadt M (2018). Hypocalcemic disorders. Best Pract Res Clin Endocrinol Metab.

[4] Munns CF, Shaw N, Kiely M (2016). Global consensus recommendations on prevention and management of nutritional rickets. J Clin Endocrinol Metab.

[5] Kiely ME (2021). Risks and benefits of vegan and vegetarian diets in children. Proc Nutr Soc.

[6] Schafer AL, Shoback DM, Feingold KR, Ahmed SF, Anawalt B (2000). Hypocalcemia: diagnosis and treatment. Endotext.

[7] Kenny CM, Murphy CE, Boyce DS (2021). Things We Do for No Reason^TM^: Calculating a “corrected calcium” Level. J Hosp Med.

[8] Khazai N, Judd SE, Tangpricha V (2008). Calcium and vitamin D: skeletal and extraskeletal health. Curr Rheumatol Rep.

[9] Abate EG, Clarke BL (2016). Review of hypoparathyroidism. Front Endocrinol.

[10] Vieira MA, Kube PK, van Helmond JL (2021). Recipe for disaster: homemade formula leading to severe complications in 2 infants. Pediatrics.

[11] Salama MM, El-Sakka AS (2010). Hypocalcemic seizures in breastfed infants with rickets secondary to severe maternal vitamin D deficiency. Pak J Biol Sci PJBS.

[12] Carvalho NF, Kenney RD, Carrington PH, Hall DE (2001). Severe nutritional deficiencies in toddlers resulting from health food milk alternatives. Pediatrics.

[13] Brusati M, Baroni L, Rizzo G (2023). Plant-based milk alternatives in child nutrition. Foods Basel Switz.

